# The potential for adaptive maintenance of diversity in insect antimicrobial peptides

**DOI:** 10.1098/rstb.2015.0291

**Published:** 2016-05-26

**Authors:** Robert L. Unckless, Brian P. Lazzaro

**Affiliations:** Department of Entomology, Cornell University, Ithaca, NY 14853, USA

**Keywords:** insect immunity, antimicrobial peptide, trans-species polymorphism, balancing selection, convergent evolution

## Abstract

Genes involved in immune defence are among the fastest evolving in the genomes of many species. Interestingly, however, genes encoding antimicrobial peptides (AMPs) have shown little evidence for adaptive divergence in arthropods, despite the centrality of these peptides in direct killing of microbial pathogens. This observation, coupled with a failure to detect phenotypic consequence of genetic variation in AMPs, has led to the hypothesis that individual AMPs make minor contributions to overall immune defence and that AMPs instead act as a collective cocktail. Recent data, however, have suggested an alternative explanation for the apparent lack of adaptive divergence in AMP genes. Molecular evolutionary and phenotypic data have begun to suggest that variant AMP alleles may be maintained through balancing selection in invertebrates, a pattern similar to that observed in several vertebrate AMPs. Signatures of balancing selection include high rates of non-synonymous polymorphism, trans-species amino acid polymorphisms, and convergence of amino acid states across the phylogeny. In this review, we revisit published literature on insect AMP genes and analyse newly available population genomic datasets in *Drosophila*, finding enrichment for patterns consistent with adaptive maintenance of polymorphism.

This article is part of the themed issue ‘Evolutionary ecology of arthropod antimicrobial peptides’.

## 1. Introduction

Genes of the immune system are among the fastest evolving in the genome. This is often attributed to an intense coevolutionary ‘arms race’ between hosts and pathogens. In this model, host populations fix a mutation conferring resistance to the pathogen, imposing selective pressure on the pathogen to re-evolve infectivity, causing the host to evolve new resistance, and the cycle repeats *ad infinitum* [1,2]. Despite the clear oversimplification of this model, molecular evolutionary data for many immune genes are consistent with such a dynamic. Immune system genes often show much more rapid adaptive evolutionary divergence than non-immune genes, indicative of recurrent bouts of selection [3–5]. Although the ‘arms race’ model is a charismatic and readily detectable mode of host–pathogen coevolution, it may not be characteristic of most immune-related genes.

Alternative models of host–pathogen coevolution can also find support in the data. For example, in time-lagged churning allele models, pathogens adapt to the most common host genotypes, driving them down in frequency [6]. Proportionally rarer alleles have a selective advantage in these models by virtue of their rarity, but they are advantageous only until they reach sufficiently high frequency for the pathogen population to adapt to them. At that point, pathogen pressure drives the allele down in frequency, in favour of different rare alleles. This allele churning is readily visible in clonal systems such as *Potamopyrgus* (a snail) infected by a trematode parasite (*Microphallus*) [7,8], but the same principle can also apply in sexually recombinant species. Importantly, such negative frequency-dependent models of evolution do not allow fixation of alleles, but instead promote allelic diversity in the host population and in some cases may result in very long-term maintenance of polymorphism [9].

The two models described above are most relevant to instances where the host is afflicted with a single dominant pathogen species. But in the vast majority of cases, hosts and pathogens are not locked in one-on-one interactions. Instead, each host species may be infected with multiple and varied pathogens, and pathogens may infect a range of hosts. In such general interactions, coevolution is ‘diffuse’ [10] and the prospects for an arms race are greatly diminished [11,12]. Furthermore, the relative abundance of pathogens or pathogen genotypes may vary in time or space, leading to fluctuation in the adaptive premiums on resistance alleles. If resistance alleles are costly in the absence of infection [13] or if alleles that confer resistance to one pathogen result in susceptibility to another [14–16], host alleles may oscillate in frequency and can be maintained for long evolutionary time [17]. Any of these instances where natural selection adaptively maintains genetic variation over time or space can be termed ‘balancing selection’ [18]. Far more difficult to detect than adaptive divergence driven by arms races, balancing selection predicts long-term preservation of ancient alleles, with genetic variation in some cases pre-dating the divergence of distinct species [19]. Of critical importance, some components of the immune system may evolve under arms races while others exhibit balancing selection, depending on the specific molecular functions of the encoded genes and the nature of interaction with pathogens.

Antimicrobial peptides (AMPs) are short antibiotic proteins produced by all plants and animals in response to infection. These peptides are secreted extracellularly and they directly kill bacteria and unicellular fungi, typically acting to permeablize or destabilize microbial membranes [20]. AMPs are generally encoded by suites of multigene families, exhibit broad specificity, and their production is often generically induced by infection and wounding. Bacterial resistance mechanisms to AMPs are similarly generic, including non-specific behaviours like secretion of proteases, efflux of AMPs from outer and inner membranes, and modification of membrane surfaces to make them less recognizable or susceptible to AMPs [21]. Thus, AMP genes may be less prone to specific coevolution with pathogens than are other components of the immune system, especially when pathogen diversity is high. Indeed, the AMP genes of invertebrates typically do not exhibit the rapid evolutionary divergence predicted under the coevolutionary arms race model, even when other components of the immune system appear to be evolving rapidly under positive selection. For example, in the genus *Drosophila*, the immune system as a whole shows a significantly higher rate of amino acid evolution than the remainder of the genome. However, genes encoding AMPs actually evolve more *slowly* than the genome average at the amino acid level [4,22]. A similar pattern is seen in the evolution of mosquito immune systems [5]. Instead, in both taxa, AMP gene families show high rates of duplication and deletion over short evolutionary timescales [4,5]. These observations led some investigators [23] to conclude that natural selection on insect AMPs is weak at the level of amino acid sequence, assuming AMPs function as efficient broad-spectrum antibiotics with little prospect for specific coevolution, and that the primary selective pressure is to maintain a minimum threshold level of expression, perhaps leading instead to selection on AMP gene expression or copy number [24,25]. This was in contrast to vertebrate AMPs, which have been more often claimed to show signs of strong positive selection or even adaptive maintenance of polymorphism [26–29]. In this article, we revisit the molecular evolution and population genetics of insect AMP genes to evaluate whether the standing interpretation that insect AMP genes experience little adaptive evolution is correct. Using a combination of new data and previously published reports, we find evidence that balancing selection and adaptive maintenance of amino acid polymorphism may be much more common in insect AMP genes than has been previously appreciated.

## 2. Material and methods

### (a) *Drosophila* sequence data

To assess population genetic patterns, we used two different strategies. First, to compare levels of polymorphism in AMPs to the rest of the genome, we used previously available estimates of polymorphism from *D. simulans* and compared AMPs to random draws from the rest of the genome. Alternatively, to examine trans-species polymorphisms (TSPs), we compared genes encoding AMPs to a set of control genes selected to match protein length and genomic position (see the electronic supplementary material, tables S1 and S2). Control genes were selected by walking out along the *D. melanogaster* genome in both directions until reaching a gene with between 100 and 800 bp of coding sequence (the approximate size of an AMP gene) that is present in at least three of the four species *D.* *melanogaster*, *D. simulans, D. mauritiana* and *D. yakuba.* Sequences for each AMP gene were obtained from publicly available datasets of *Drosophila simulans* (*n* = 20 [30]) and *D. yakuba* (*n* = 20 [30]), *D. mauritiana* (*n* = 10 [31]) and African *D. melanogaster* (DPGP3 [32]). To achieve equivalent sample sizes, we downsampled the *D. melanogaster* DPGP3 sequences by randomly selecting 20 sequences from the larger sample of 197. Of the four species, only *D. simulans* and *D. mauritiana* are thought to interbreed to any extent [33]. Sequences were aligned using ClustalW [34], then checked, and alignments were corrected by eye as necessary. Simple neighbour joining trees were constructed in Geneious [35] using a Jukes–Cantor distance method. DNA sequences were translated to amino acids and amino acid sequences were realigned. DNA sequence alignments were deposited in Dryad, accession no. 10.5061/dryad.g8n25.

### (b) DNA polymorphism and divergence

To assess levels of non-synonymous polymorphism and divergence in *D. simulans* AMPs compared to the rest of the genome, we used estimates from publicly available whole-genome sequencing of seven *D. simulans* inbred lines [36]. Per site non-synonymous polymorphism (*π_a_*) and unpolarized non-synonymous divergence (*d_n_*) were recorded for each AMP, and the mean for AMPs was compared to a null distribution of 10 000 random draws of size 28 (the number of AMP genes evaluated) from all genes. In this analysis, we employed whole-genome data, not a control set of genes. We also compared the ratio of non-synonymous polymorphism to non-synonymous divergence (*π_a_/d_n_*) for AMPs to the distribution of random draws. Non-synonymous divergence was increased by a factor of 0.0001 (the minimum non-zero value) for all genes to avoid zeros in the denominator. We do not report analyses based on synonymous polymorphism linked to putatively selected sites because we suspect that high rates of effective recombination may in many cases erode signatures of linked selection [37].

### (c) Trans-species polymorphisms

TSPs were identified by visually inspecting amino acid alignments. Any site segregating for the same two amino acids in more than one species was designated a TSP. Given the small sample size, we did not employ a minor allele frequency cut-off.

Differences in the number of TSPs between AMPs and our control set were assessed individually for each species pair using logistic regression with state (co-segregating or non-co-segregating) as a binary response variable and class (AMP or control) as a factor. This analysis was conducted using the glm function in R [38].

TSPs in AMP genes from non-*Drosophila* insect species were identified from sequences deposited into Genbank after alignment and translation as described in §2a.

### (d) Molecular evolutionary convergence

We examined amino acid convergence across the genus *Drosophila* as a means of identifying potential instances of parallel evolution by natural selection. Evolutionary convergence in the genome reference sequences for 22 species was assessed using alignments pulled from the UCSC genome browser [39]. We considered polymorphic sites in *D. melanogaster*, *D. simulans*, *D. mauritiana* and *D. yakuba*, but all other species were represented by a single haploid sequence from an inbred line. Sequences were compiled and aligned as described in §2a. For each amino acid variant in the alignment, a simple convergence score was defined as the minimum number of independent mutations on the phylogeny at that position, minus the number of observed amino acid states, plus one. For example, if two non-sister species have serine at a given residue and all other species have glycine, there are two independent mutations (on the lineages leading to each of the non-sister species) and two states (serine and glycine), so we infer one (2 – 2 + 1) instance of convergence over the phylogeny. We assessed enrichment of convergences at sites with TSPs by comparing the number of convergences at those sites to sites with polymorphisms private to *D. melanogaster*. Statistical significance was assessed using a Mann–Whitney *U*-test implemented in R [38].

## 3. Results

Our analysis was motivated by a recent study in which we characterized balancing selection acting on the gene encoding the *Drosophila* AMP, Diptericin [37]. In that work, we noted that a naturally occurring non-synonymous mutation from serine to arginine at the 69th position of the Diptericin mature peptide resulted in a substantial decrease in resistance to bacterial infection in both *D. melanogaster* and *D. simulans*. These two species are not sister taxa, and they have independently derived the arginine state via different mutations of the 69th codon. At the site of the serine/arginine polymorphism, we observed five independent transitions to arginine across 24 *Drosophila* species, suggesting that a common selective pressure might promote the use of arginine at this position in diverse species. Some *D. simulans* chromosomes carry a tandem duplication of the *Diptericin* gene and the serine/arginine polymorphism segregates in both paralogues. Based on the entire body of evidence, we argued that the serine/arginine polymorphism in *Diptericin* is likely to be adaptively maintained through fluctuation in environment and/or pathogen pressure across time and/or space. In this work, we ask whether the qualitative pattern observed in the *Diptericin* locus is generic to AMP genes, and whether insect AMPs may evolve under balancing selection more often than is generally appreciated.

We focus our new data analysis on the AMP genes of *Drosophila*, as these have been the most extensively studied group of AMPs and *Drosophila* species provide the greatest depth in population genomic data among insects. We (i) compare amino acid polymorphism and divergence in AMPs to the rest of the genome in *D. simulans*, (ii) scan AMPs for TSPs and compare their rate of occurrence to the rate observed in a set of control genes in several species pairs, and (iii) measure the excess rate of evolutionary convergence at TSPs relative to polymorphisms private to *D. melanogaster*. We use the aggregate information to infer whether AMP genes as a class are more likely to evolve under balancing selection than are matched control genes drawn from the background genome.

### (a) Excess polymorphism in *Drosophila* antimicrobial peptides

Genes under long-term balancing selection are expected to exhibit more synonymous and non-synonymous polymorphism than those evolving neutrally, because the comparatively old age of the selectively balanced mutation (deep coalescent) permits the accumulation of mutations on the genealogical lineage that separates the balanced alleles [19]. We compared non-synonymous nucleotide diversity (*π_a_*) and unpolarized non-synonymous divergence (*d_n_*) using previously published data from *D. simulans* AMPs [36] to an empirical distribution constructed by sampling groups of 28 genes from the genome.

**Figure 1.**
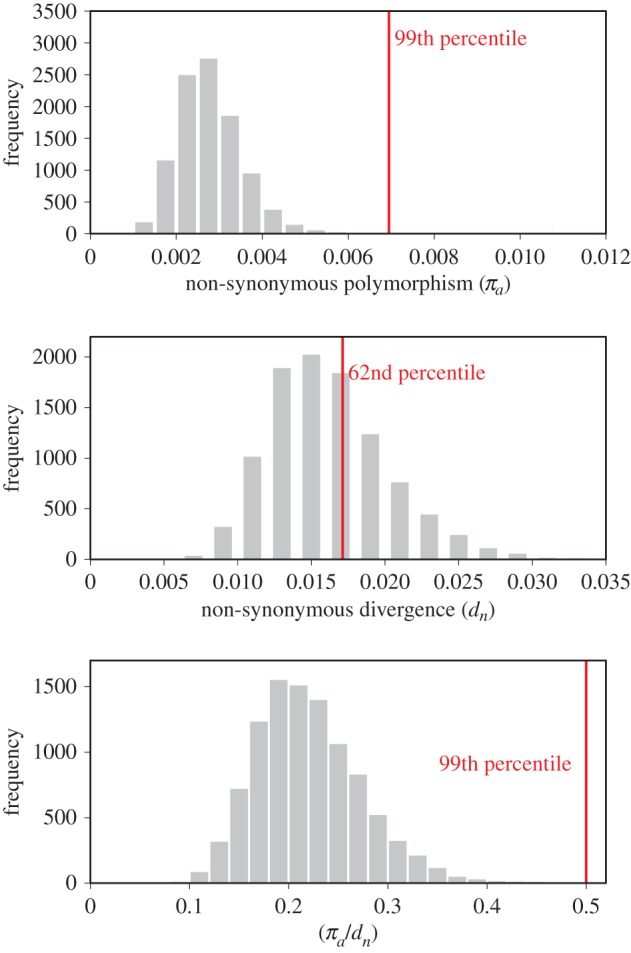
Mean non-synonymous diversity (*π_a_*), divergence (*d_n_*) and the ratio of the two (*π_a_*/*d_n_*) in AMPs indicated by a vertical red line over the distribution of mean values from 10 000 draws of control gene sets sampled from the genome.

The mean non-synonymous nucleotide diversity for AMPs was in the 99th percentile of randomly sampled sets of genes, indicating that AMPs exhibit exceptional amino acid polymorphism compared to the rest of the genome (figure 1; electronic supplementary material, table S3). High rates of non-synonymous diversity could arise through either balancing selection or relaxed constraint on protein sequences. If AMP sequences are comparatively unconstrained, we would also predict higher than average non-synonymous divergence between species in AMPs compared with the rest of the genome. Yet AMP non-synonymous divergence is not in the tail of the genome-sampled distribution (figure 1, 62nd percentile). We computed the ratio of non-synonymous polymorphism to divergence for AMPs and the resampled genome sets. Once again, AMPs were exceptional (99th percentile) for the highest ratios of non-synonymous polymorphism to divergence. Thus, we conclude that the excess in AMP genes polymorphism is unlikely to be due to relaxation of selective pressures leading to a generally higher rate of selectively neutral substitution. The data are more consistent with population-level maintenance of variation that does not fix in the species.

### (b) Evidence of trans-species polymorphism in antimicrobial peptides

Ancient balanced polymorphisms can segregate in multiple closely related species if they pre-date the species split [40]. Polymorphisms can also be shared between closely related species via interspecific hybridization and introgression [41]. Even distantly related species can share polymorphism if the same variants arise through independent mutation (e.g. [37]). Finally, independent paralogous gene conversion within two species can result in TSP when both species harbour closely related paralogues.

*Drosophila* AMP genes are rife with TSPs (table 1). Many of these are found in the signal peptide or pro-peptide regions. For example, between *D. melanogaster* and *D. simulans*, we find nine TSPs spread over six different AMPs (out of 11 examined). This is a species pair with no evidence of ongoing introgression and whose divergence time (approx. 3–5 Ma [42]) far exceeds expected time for which selectively neutral variation from the common ancestral species is expected to persist (4*N*_e_ generations where *N*_e_ is the effective population size, or approximately 400 000 generations or 25 000 years [43]). Other species pairs show similar significant excesses of TSP in AMP genes relative to control genes, although the more divergent *D. yakuba* is less likely to harbour TSPs with the ingroup species (table 1; electronic supplementary material, tables S4 and S5).

**Table 1.** Trans-species amino acid polymorphisms in AMPs. Reported as A1PA2-Dp, where P is position in alignment, A1 and A2 are the two amino acid alleles, D is the domain (s = signal peptide, p = pro-domain, m = mature peptide; for Attacin D, n = N-terminal, c = C-terminal) and p is the position in the domain. n.d., no comparison due to poor alignments.

AMP	*length*	*mel-sim*	*mel-mau*	*mel-yak*	*sim-mau*	*sim-yak*	*mau-yak*
*Attacin A*	236	L21V-p1 A39T-m8	L21V-p1	n.d.	L9M-s9^b^ L21V-p1	n.d.	n.d.
*Attacin B*	236	L18V-p1 A36T-m8	L18V-p1	n.d.	L18V-p1	n.d.	n.d.
*Attacin C*	244	—	T28N-p7	—	—	—	—
*Attacin D*	181	F82I-c16	—	—	A40ST-n40 A58G-n58 M69V-c3 L151V-c85	—	—
*Cecropin A*^c^	64	N2K-s2	T17S-s17(A2)^a^	A17T-s17(A2)	—	—	—
*Cecropin B*	63	—	—	A15V-s15	M10V-s10	—	—
*Defensin*	92	—	M17V-sp17	—	—	H41Q-p20	—
*Diptericin A*^d^	106	S92R-m69(A1,A2)^a^	—	—	—	—	—
*Diptericin B*	—	—	A19V-sp19	—	—	—	—
*Drosocin*	64	A52T-p12	—	—	—	—	—
*Metchnikowin*	53	P50R-m24	P50R-m24	P50R-m24	P50R-m24	P50R-m24	P50R-m24

^a^Convergent mutations.

^b^Also valine in *D. simulans.*

^c^*D. melanogaster CecA1* and *CecA2* considered.

^d^*D. simulans DptA1* and *DptA2* considered.

A few specific examples are worth noting. We previously reported two instances of convergence at *Dpt* in *D. melanogaster* and *D. simulans* [37]. First, serine (hydrophobic, neutral) and arginine (positively charged) are segregating at the 69th position of the mature peptide via independent mutation in both *D. melanogaster* and *D. simulans*. Second, convergent deletions eliminate the *Diptericin* start codon in some alleles of both species, presumably rendering the gene non-functional. Both of these convergences have measurable phenotypic impact on resistance to bacterial infection [37].

In a previous study, Lazzaro & Clark [44] noted that alanine and threonine (both hydrophobic) are segregating at the 12 position in the pro-peptide sequence of *Drosocin* in *D. melanogaster*. Lazzaro & Clark [44] noted a significant increase in the frequency of the ancestral alanine allele between 1998 and 2001 with markedly reduced diversity in chromosomes carrying the alanine state, suggesting a partial selective sweep favouring the ancestral allele. In a subsequent survey of haphazard natural collections taken between 2008 and 2014, the allele frequencies continue to match those observed in 2001 (SM Rottschaefer & BP Lazzaro 2015, unpublished data). The fact that the derived threonine allele also segregates in *D. simulans* (employing the same codon sequence) is intriguing, although the threonine state is rare in *D. simulans* (0.05% observed frequency).

In the Attacin A/B locus, a lack of reciprocal monophyly between *D. simulans* and *D. mauritiana* is suggestive of introgression through hybridization between species (electronic supplementary material, figure S1). However, a systematic study of introgression patterns between the two species [33] did not report any of the Attacin genes as having a signature of recent introgression, indicating that, if these regions are introgressed, either the introgression was ancient and this polymorphism has persisted or the introgressed region was too small to be classified as such in the previous study. Lysine (positive charge) and valine (hydrophobic/aliphatic) are segregating in both *Attacin A* and *Attacin B* in *D. melanogaster, D. simulans* and *D. mauritiana* at the first residue of the pro-peptide (position 21 in *AttA* and position 18 in *AttB* since a three amino acid insertion in *AttA* shifts the alignment). Lazzaro & Clark reported gene conversion between paralogues in *D. melanogaster* [45], and it stands to reason that paralogous gene conversion may occur in other species as well. For non-adaptive processes to be responsible for this sharing of polymorphism between paralogues in three species, we have to posit that the same mutations arose, were shared between paralogues through independent gene conversion in three species, and have drifted to intermediate population frequency in all of them. This seems unlikely. Similarly, we find alanine and threonine segregating in both *Attacin A* and *Attacin B* at the eighth position of the mature peptide in both *D. melanogaster* and *D. simulans*. Paralogous gene conversion may facilitate maintenance of conditionally beneficial genetic variation since that variation can be regenerated from the paralogous copy.

Finally, and perhaps most interestingly, proline (uncharged) and arginine (positively charged) segregate in the third-to-last position of the *Metchnikowin* mature peptide of *D. melanogaster* (80% proline, *n* = 20), *D. simulans* (52% proline, *n* = 21), *D. mauritiana* (20% proline, *n* = 10) and *D. yakuba* (90% proline, *n* = 20). In all species, the same mutation is responsible for the amino acid change (C to G in the middle position of the codon, the only single mutation that would result in a change from arginine to proline) so we cannot unambiguously determine whether these mutations are convergent or have been maintained since before the divergence of these four species.

To systematically assess whether *Drosophila* AMPs harbour an excess of TSPs relative to the genome expectation, we contrasted the relative incidence of TSPs in AMP genes relative to control genes using logistic regression (see §2c). AMPs harboured significantly more TSPs than control genes in one of six pairwise comparisons (*D. melanogaster/D. simulans*, *D. melanogaster/D. mauritiana* and *D. melanogaster/D. yakuba* were nearly significant) and harboured a non-significant excess in two of the remaining comparisons (electronic supplementary material, table S5). Using Fisher's method for combining *p*-values, we find a nearly significant excess of TSPs in AMPs compared to our control set (*p* = 0.055). The AMPs have a similar number of TSPs per polymorphic amino acid residues as the control genes do (electronic supplementary material, table S1), indicating that the excess of TSPs in AMPs is at least partially related to their overall increased amino acid diversity. A similar pattern was observed genome-wide by Begun *et al.* [33], who reported a higher rate of transpecific amino acid polymorphisms when amino acid diversity was the highest.

### (c) Convergence on the *Drosophila* phylogeny

**Figure 2.**
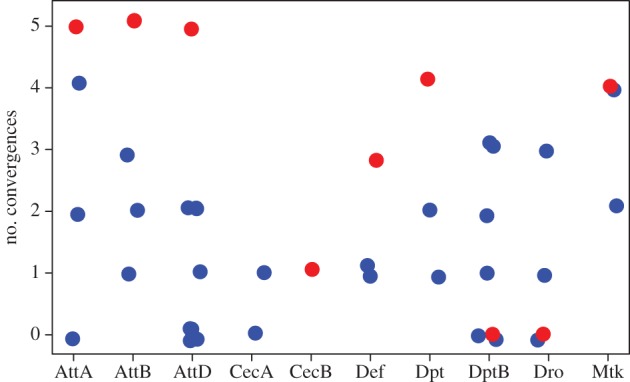
Number of convergences on the *Drosophila* phylogeny for TSPs (red) and amino acid polymorphisms private to *D. melanogaster* (blue).

An excess of transitions between two amino acid states in multiple independent species lineages could indicate that selection favours both alleles in alternative environments. To assess whether TSPs show more instances of convergence across the genus *Drosophila*, we tested whether TSPs are more likely to convergently re-evolve elsewhere on the tree than are amino acid polymorphisms private to *D. melanogaster*. TSPs showed higher rates of convergence than polymorphisms that were private to *D. melanogaster* (Mann–Whitney *U* = 78; *p* = 0.014; figure 2). The median number of convergences for TSPs was four (range from zero to five) compared to a median of one (range from zero to four) for *D. melanogaster* private polymorphisms. For most of the species on the tree, we have only a single reference genome sequence. It thus remains possible that with deeper sampling, the convergences could be revealed to be polymorphic in additional species. This excess of convergence at sites with TSPs is consistent with either bias towards neutral substitution of the same amino acids at TSP positions, or an adaptive value that maintains the two or more alternative states at each position. Given that the one mutation we have best characterized, the serine/arginine polymorphism in the mature peptide of *Diptericin*, has a large phenotypic effect on resistance [37], we have a hard time envisioning that these recurrent convergent substitutions are adaptively neutral. Instead, we favour the hypothesis that geographical and/or temporal variation in selective forces such as infectious pathogen pressure leads to adaptive maintenance of polymorphism within species and recurrent convergent evolution across species.

We have identified multiple new and potentially interesting amino acid substitutions that not only segregate in multiple species but also arise by convergence elsewhere on the phylogeny (electronic supplementary material, figure S2). For example, *Mtk* is segregating for arginine and proline at position 50 of the mature peptide in *D. mauritiana, D. simulans, D. melanogaster* and *D. yakuba*. Across the phylogeny, we find two additional mutations to arginine at this position: one in *D. erecta* and another in *D. kikkawai.* There are also two mutations to glycine at the same position (one in the branch leading to *D. biarmipes, D. suzukii* and *D. takahashii* and another in *D. elegans*)*.* The derived arginine allele segregating in *Diptericin A* position 69 in *D. melanogaster* and *D. simulans* is also found in the reference sequences for *D. orena*, *D. ficusphila* and *D. willistoni* [37]. Additionally, there are two mutations to glutamine at the same site. Other striking instances of convergence include the two polymorphisms in *Defensin*, those in *Attacin D* and one in the mature peptide of *Attacin A/Attacin B* (electronic supplementary material, figure S1)*.*

### (d) Revisiting antimicrobial peptide evolution in other insect taxa

Few previous studies in non-*Drosophila* insects have generated data that can be screened for the presence of TSPs, convergence over a phylogeny, or even balancing selection on AMPs. Below we revisit the subset of existing studies from non-*Drosophila* insects with the most appropriate data for addressing these questions, and we find several instances of TSP in other taxa.

Termicin is a termite Defensin protein. Two studies examined polymorphism in *termicin* in four species [46,47]. Re-examining this polymorphism data, we find valine and arginine segregating at the 13th position of the signal peptide and histidine and arginine segregating at the 14th position of the mature peptide in *Reticulitermes chinensis* and *Odontotermes formosanus.* Neither of these polymorphisms show convergence over the remainder of the termite phylogeny [48].

Alanine and valine segregate in the sixth position of the signal peptide of Cecropin E of the silkworms *Bombyx mori* (*n* = 15) and *Bombyx mandarina* (*n* = 12) [49]. Examination of *Defensin A* and *moricin* from these same samples revealed no non-synonymous TSPs, because there was no amino acid polymorphism in *B. mori* in either of these genes. No closely related species have been sequenced to allow a test of convergence across the *Bombyx* phylogeny.

Glutamine and arginine segregate at the 86th position of the mature peptide in Hymenoptaecin of the ant, *Monomorium cyaneum* [50] and the bee, *Apis cerana* [51]*.* Comparisons of other AMPs in hymenopterans were not possible due to poor alignments or only single-species polymorphism data.

AMPs in *Anopheles* mosquitoes harbour frequent TSPs, although this may be due in part to ongoing gene flow between named species [52–54]. Both phenylalanine and isoleucine are segregating at the eighth position of the signal peptide of Cecropin A in *Anopheles gambiae* and *Anopheles melas*—two species unlikely to have experienced recent introgression. Several AMPs show TSP between *A. gambiae* and *A. arabiensis*, although these species are very recently diverged, still sharing polymorphism in many genes and probably hybridizing in the field [55]. In Defensin, both alanine and serine segregate at the 15th position of the signal peptide in *A. gambiae* and *A. arabiensis*, and both alanine and serine segregate at the 12th position of the mature in *A. gambiae* and *A. arabiensis.* In Gambicin, the fourth position of the signal peptide segregates for methionine and valine in *A. gambiae* and *A. arabiensis* and the seventh position of the signal peptide segregates for leucine and isoleucine in the same two species. In both *A. arabiensis* and the more distantly related *A. merus*, the 37th position of the Gambicin mature peptide segregates for alanine and threonine. Phenylalanine and valine segregate at the 55th position of the mature peptide in *A. arabiensis, A. gambiae* and the equally closely related *A. quadriannulatus.*

Evaluation of the available data from multiple insects indicates that the observation of TSP in AMP genes is not unique to *Drosophila*. In the absence of population genomic sequencing to yield a set of control genes, we cannot assess whether TSP is more common in AMPs than in other genes in these species. Nevertheless, the existing data suggest that TSP in AMP genes may be widespread, and we predict that molecular convergence in more distantly related species is probably also common. These predictions can be tested as high-throughput sequencing enables population genomic data collection from a wide variety of insect species.

## 4. Discussion

Prevailing wisdom has been that insect AMPs function as an antibiotic cocktail, where the component proteins have distinct but partially redundant mechanisms of attacking the microbe. This functional hypothesis combined with little evidence of adaptive diversification in insect AMPs led to a general theory that collective AMP dosage is the primary determinant of resistance, and that individual AMP genes are under little natural selective pressure (e.g. [23]). This interpretation was in contrast to observations from vertebrates, where adaptive maintenance of polymorphism has been suggested for AMPs in humans [29,56], birds (including TSP) [28] and frogs [27]. More recently, TSP and adaptive maintenance of polymorphism have been additionally observed in marine mussels [57,58].

Also in contrast to the prevailing wisdom about insect AMPs, two recent studies in *Drosophila* have shown that deletion of a single or small number of AMP genes can have major impact on organism-level resistance to bacterial infection [59,60]. At the *Diptericin* gene, a single amino acid polymorphism is strongly predictive of resistance [37], suggesting that natural selection could act effectively on individual AMP genes. Although there is little evidence in insect AMPs of adaptive divergence in the sense of ‘arms races’, a re-reading of previous literature shows many patterns consistent with potential balancing selection (table 2), including several instances that went undiscussed by the authors in early *Drosophila* papers [44,45,61,62]. We propose a few explanations for this absence. First, many studies were conducted prior to 2005, and balancing selection was generally given less consideration by population geneticists because few formal tests existed. Second, AMPs are small and therefore most standard tests are underpowered—especially if recombination has broken down haplotypes associated with the maintained alleles. Finally, it is only recently that genome-scale analyses and very large sample sizes have become accessible to most researchers, and these may be very important for detection of possible balancing selection. With the present analysis, we suggest that adaptive maintenance of polymorphism and convergent evolution may be more common in insect AMP genes than was previously recognized. Both balancing selection and convergent evolution can be driven by fluctuation in natural selective pressure over time and/or geographical space, and may be mediated by shifting diversity of pathogens as well as by correlated life-history costs of overactive immune systems [19,70]. Patterns of AMP evolution may be further influenced by synergistic interactions among distinct AMPs (e.g. [71,72]), especially if the synergism is quantitatively altered by amino acid changes to the interacting AMPs. We strongly advocate more targeted research to investigate the frequency of adaptive AMP polymorphism in insects that are amenable to experimental study.

**Table 2.** Summary of evidence for selection in AMPs. TSPs = trans-species polymorphisms (table 1)—each instance is counted so a polymorphism present in all four species is counted six times for six comparisons. n.d.=Not determined.

taxon	AMP	TSPs	conv. on phylogeny	positive selection^a^	other patterns consistent with balancing selection
*Drosophila*	*AttA*/*AttB*	9	several	no—*AttA* *AttB*—negative Tajima's *D* [45]	gene conversion between paralogues [45]
	*AttC*	1	very little	no [45]	—
	*AttD*	5	moderate	n.d.	—
	*CecA*	3	very little	no [61,62]	high *F*_st_ between populations [61] positive Tajima's *D* [62]
	*CecB*	2	none	no [61,62]	high *F*_st_ between populations [61]
	*Def*	2	several	no [44]	—
	*Dpt A*	1	several	negative Fu and Li's *D* [61] no [44]	high *F*_st_ between populations [61] phenotypic evidence [59]
	*Dpt B*	1	none	no [44]	—
	*Dro*	1	none	no [44]	—
	*Mtk*	6	several	no [44]	—
*Bombus*	*Def-1*	n.d.	n.d.	high d*N*/d*S* [63]	—
	*Abaecin*	n.d.	n.d.	high d*N*/d*S* [63] no [64]	—
	*Hymenoptaecin*	n.d.	n.d.	high d*N*/d*S* [63]	—
*Apis mellifera*	*Def-2*	n.d.	n.d.	negative Fu and Li's *D*^a^ and negative Fu and Li's F^a^ [65]	—
	*Lys-1*	n.d.	n.d.	negative Fu and Li's *D*^a^ and negative Fu and Li's F^a^ [65]	high non-synomymous polymorphism with low divergence
*Reticulitermes*	*Termicin*	2	n.d.	significant MK test [47]	—
*Anopheles*	*Gambicin*	4	n.d.	PAML—positive selection on single codon [54] no [51]	positively selected site based on PAML analysis is segregating in three species
	*Defensin*	2	n.d.	purifying selection [54,66]	—
	*Cecropin 1*	1	n.d.	no [67]	
*ants*	*Defensin*	n.d.	yes [68]	PAML—positive selection acting on two codons [68]	—
*Ixodes*	*Defensin*	n.d.	n.d.	negative Tajima's *D*, negative Fu and Li's F [69]	very polymorphic gene, but polymorphism confined to intron
*Bombyx*	*Defensin A*	no	n.d.	negative Tajima's *D* [49]	high ratio of non-synonymous to synonymous polymorphism in *B. mandarina*

^a^Sackton *et al.* [4] and Obbard *et al.* [22] found no evidence for adaptive evolution in any *Drosophila* AMPs.

We emphasize that TSP and evolutionary convergence do not definitively prove maintenance of polymorphism by balancing or fluctuating selection. However, we do see several patterns that are consistent with balancing selection and that are more common in AMP genes than in the background genome. These include high rates of non-synonymous polymorphism, TSP, amino acid convergence across phylogenetically distinct taxa and high incidence of loss of function alleles. Although these patterns could also be consistent with relaxed selective constraint, we are wary of that explanation because the amino acid substitutions are often non-conservative and because at least some functionally tested alleles have clear phenotypic effects.

Unfortunately, there are some deficiencies in the available data that preclude a more definitive analysis of whether insect AMP genes systematically evolve via maintenance of polymorphism. First, we lack well-annotated arthropod genomes outside of *Drosophila*. Even in *Drosophila*, the majority of AMPs are not functionally characterized and whole new families may yet be discovered, exemplified by the novel *Bomanin* family described in 2015 [60]. Poor genome assembly is especially a problem for analysis of multigene families, where inference of shared polymorphism and convergence can be confounded, but ambiguity in orthologous relationships. This problem is exacerbated when the sequence data are generated by short-read technology. Second, we lack population genomic datasets for pairs or groups of species. Such datasets are beginning to be generated, but most datasets to date involve pairs of species that are so closely related that they continue to hybridize and exchange alleles. This muddies the interpretation of trans-specific polymorphism and retention of ancestral variation. Finally, because AMP genes are short, they harbour little nucleotide diversity, reducing the power of many population genetic tests on individual genes. Thus, empowered analyses rely on pooling the data from multiple AMP genes, which may vary in precise function, activity and tissue of expression. An understanding of the selective forces acting on AMPs also requires a better understanding of the functional consequences of amino acid variation. Both *in vitro* and *in vivo* assays will assist in the functional dissection of natural variation in the mature peptide as well as signal and pro domains of AMPs. This functional understanding may even illuminate drivers for similar patterns of AMP diversity in ecologically diverse host species.

Despite the limitations in the available data, we propose that there is credible and suggestive evidence that balancing selection and adaptive maintenance of polymorphism may be common in insect AMP genes. We have highlighted some of these lines of evidence, and advocate targeted experimentation in diverse insects to formally test the hypothesis. These experiments should take the form of both population genomic analyses and phenotypic study, and they are well within the reach of the research community. The first 10 years of research on the evolution of insect AMP genes led to the conclusion that individual AMPs are superfluous and redundant, and largely invisible to natural selection. Will the next 10 years overturn that interpretation?

## Supplementary Material

Supplementary Material

## Data accessibility

All data were previously published, and where appropriate is present in the electronic supplementary material.
