# Supplementary material for: The potential for adaptive maintenance of diversity in insect antimicrobial peptides
Source: Philos Trans R Soc Lond B Biol Sci. 2016 May 26;371(1695):20150291. doi: 10.1098/rstb.2015.0291 (PMC4874389; doi:10.1098/rstb.2015.0291)
Supplement: Supplementary Material [file rstb20150291supp1.pdf]

Unckless RL and Lazzaro B 2016. The potential for adaptive maintenance of diversity in insect antimicrobial peptides. *Phil. Trans. R. Soc. B.* doi:10.1098/rstb.2015.0291

Supplementary material

Table S1. Counts of TSPs and polymorphisms (that are not TSPs) in AMP and control genes.

				D. melanogaster/ D. simulans		D. melanogaster/ D. mauritiana		D. melanogaster/ D. yakuba		D. simulans/ D. mauritiana		D. simulans/ D. yakuba		D. mauritiana/ D. yakuba	
**Gene**	**Category**	**Coordinates**	**Length (bp)**	**TSP**	**Other**	**TSP**	**Other**	**TSP**	**Other**	**TSP**	**Other**	**TSP**	**Other**	**TSP**	**Other**
Mtk	AMP	2R:15,408,846..15,409,113 [+]	162	1	7	1	2	1	10	1	7	1	14	1	8
Def	AMP	2R:10,054,178..10,054,576 [-]	279	0	13	1	4	0	4	0	12	1	10	0	3
Dro	AMP	2R:14,745,961..14,746,714 [+]	195	1	8	0	4	0	10	0	6	0	12	0	6
CecB	AMP	3R:30,213,315..30,213,797 [-]	192	0	6	0	3	1	4	1	4	0	9	0	6
CecA	AMP	3R:30,210,874..30,211,273 [+]	192	1	5	1	3	1	5	0	4	0	7	0	4
DptB	AMP	2R:18,867,391..18,867,895 [+]	363	0	10	1	15	0	11	0	13	0	7	0	11
AttA	AMP	2R:14,747,362..14,748,215 [+]	663	2	21	1	19	NA	NA	2	10	NA	NA	NA	NA
AttB	AMP	2R:14,749,223..14,750,165 [+]	657	2	12	1	14	NA	NA	1	14	NA	NA	NA	NA
AttC	AMP	2R:13,393,705..13,394,667 [+]	734	0	20	1	19	0	18	0	21	0	18	0	20
AttD	AMP	3R:17,625,268..17,626,086 [+]	546	1	37	0	27	0	11	4	35	0	29	0	20
Dpt	AMP	2R:18,865,765..18,866,260 [+]	319	1	6	0	3	0	8	0	6	0	9	0	6
CG31441	Control	3R:11,236,286..11,237,624 [+]	147	0	5	0	4	0	1	0	6	0	4	0	3
CG8204	Control	2R:15,573,144..15,573,991 [-]	432	1	27	1	14	0	8	2	30	0	23	1	19
ave	Control	2R:14,752,521..14,753,269 [+]	21	0	1	0	1	0	0	0	2	0	1	0	0
CG12861	Control	2R:14,664,246..14,665,356 [-]	720	0	5	0	7	0	5	0	6	0	4	0	6
CG15107	Control	2R:18,971,309..18,972,537 [-]	741	0	17	0	14	0	24	0	16	1	23	0	23
CG33998	Control	2R:18,738,492..18,739,143 [+]	360	1	7	0	9	1	9	1	7	0	9	0	8
CG15863	Control	2R:9,959,119..9,959,732 [-]	399	0	7	0	3	0	4	0	8	1	7	0	5
Sec61Beta	Control	2R:14,618,771..14,619,681 [+]	303	0	1	0	1	0	1	0	0	0	0	0	0
CG15529	Control	3R:30,152,059..30,153,699 [+]	591	0	12	0	14	0	17	2	10	0	13	0	15
CG15526	Control	3R:30,055,957..30,056,603 [+]	342	0	13	0	6	0	7	0	11	0	12	0	6
mRpS18C	Control	3R:30,423,974..30,424,556 [+]	423	0	8	0	7	0	10	0	7	0	10	0	9
CG30065	Control	2R:13,433,028..13,433,760 [+]	141	0	7	NA	NA	0	7	NA	NA	0	6	NA	NA
CG31360	Control	3R:17,685,097..17,685,807 [+]	297	0	5	1	5	0	3	0	7	0	3	1	4
Logistic regression P				0.073		0.201		0.239		0.472		1		0.655	

TSP=trans-species polymorphism

Other=Other polymorphic amino acid residues (in one or both species)

Chr=chromosome

Coordinates=chromosome coordinates from FlyBase release 6 (FB2015_05) for *D. melanogaster*; +/- represent strand

Table S2. Weighted mean and standard deviation (SD) of synonymous divergence in the genes used for TSP analysis for each species pair.

**Species 1**	**Species 2**	**Mean**	**SD**
D. melanogaster	*D. simulans*	0.144	0.058
D. melanogaster	*D. yakuba*	0.332	0.091
D. melanogaster	*D. mauritiana*	0.147	0.052
D. simulans	*D. Yakuba*	0.301	0.099
D. simulans	*D. mauritiana*	0.053	0.045
D. Yakuba	*D. mauritiana*	0.308	0.091

Table S3. Nonsynonymous polymorphism and divergence in *D. simulans* AMPs from Begun *et al.* 2007.

Gene Name	Chr	Position	π_a	d_n
AttA	2R	14747391	0.0057	0.0251
AttB	2R	14749255	0.0051	0.0052
AttC	2R	13393769	0.0015	0.0102
AttD	3R	17625324	0.022	0.0192
IM1	2R	18384023	0.0103	0.0153
CG18107	2R	18384637	0.004	0.0393
IM2	2R	18386673	0.0051	0.0128
IM3	2R	18388185	0	0.0121
CG15065	2R	18389989	0.06	0.0404
CG15068	2R	18390395	0	0.0226
CG16836	2R	18389100	0.0256	0.0497
IM23	2R	18382789	0.0046	0.0321
CG15067	2R	18385055	0.0015	0.0126
CG5791	3R	22087203	0.0023	0.0154
CecA1	3R	30210948	0.0056	0.0099
CecB	3R	30213479	0	0.007
CecC	3R	30216577	0	0
Def	2R	10054292	0.0019	0.0153
DptB	2R	18867422	0.0056	0.0159
Dro	2R	14745990	0.0087	0.0205
Drs	3L	3369619	0	0
Drsl-1	3L	3335582	0	0.0358
Drsl-2	3L	3314375	0.0031	0.0015
Drsl-3	3L	3315034	0.0062	0.0348
Drsl-4	3L	3315637	0	0
Drsl-5	3L	3316835	0	0
Drsl-6	3L	3336147	0.0042	0.021
Mtk	2R	15408876	0.0116	0.0058

Chromosome positions refer to release 6 (FB2015_05) in *D. melanogaster*.

Table S4. Trans-species polymorphisms in control genes. Reported as PA1A2 where P is position in alignment, A1 and A2 are the two amino acid alleles. ND – no comparison due to poor alignments.

Gene	*mel-sim*	*mel-mau*	*mel-yak*	*sim-mau*	*sim-yak*	*mau-yak*
CG31441	-	-	-	-	-	-
CG8204	R12K	A31T	-	E71T<br>A139T	-	N47K
ave	-	-	-	-	-	-
CG12861	-	-	-	-	-	-
CG15107	-	-	-	-	I190L	-
CG33998	A14T	-	T89I	K87Y	-	-
CG15863	-	-	-	-	M88L	-
Sec61β	-	-	-	-	-	-
CG15529	-	-	-	G47D<br>60SP	-	-
CG15526	-	-	-	-	-	-
MRPS18C	-	-	-	-	-	-
CG30065	-	ND	-	ND	-	ND
CG31360	-	E81K	-	-	-	S100F

Table S5. Trans-species polymorphisms (TSPs) summed across all genes for AMPs and a control set. P-values assessed using logistic regression (see methods). P-value for total significance based on Fisher's method for combining P-values.

Constrast	AMP TSPs	Control TSPs	P-value
D. melanogaster/D. simulans	9/1459	2/1771	**0.012**
D. melanogaster/D. mauritiana	7/1459	2/1771	0.053
D. melanogaster/D. yakuba	3/987	1/1771	0.110
D. simulans/D. mauritiana	9/1459	5/1771	0.177
D. simulans/D. yakuba	2/987	2/1771	0.561
D. mauritiana/D. yakuba	1/987	2/1771	0.897
**Total**	32/7338=0.004	14/10626=0.001	0.055

Figure S1. Bootstrapped Neighbor-Joining tree of *Drosophila Attacin A* (circles) and *Attacin B* (squares) sequences. Colors represent: black=*D. yakuba*, green=*D. simulans*, blue=*D. mauritiana* and orange=*D. melanogaster*. Node labels are bootstrap values for nodes with better than 50% bootstrap support.

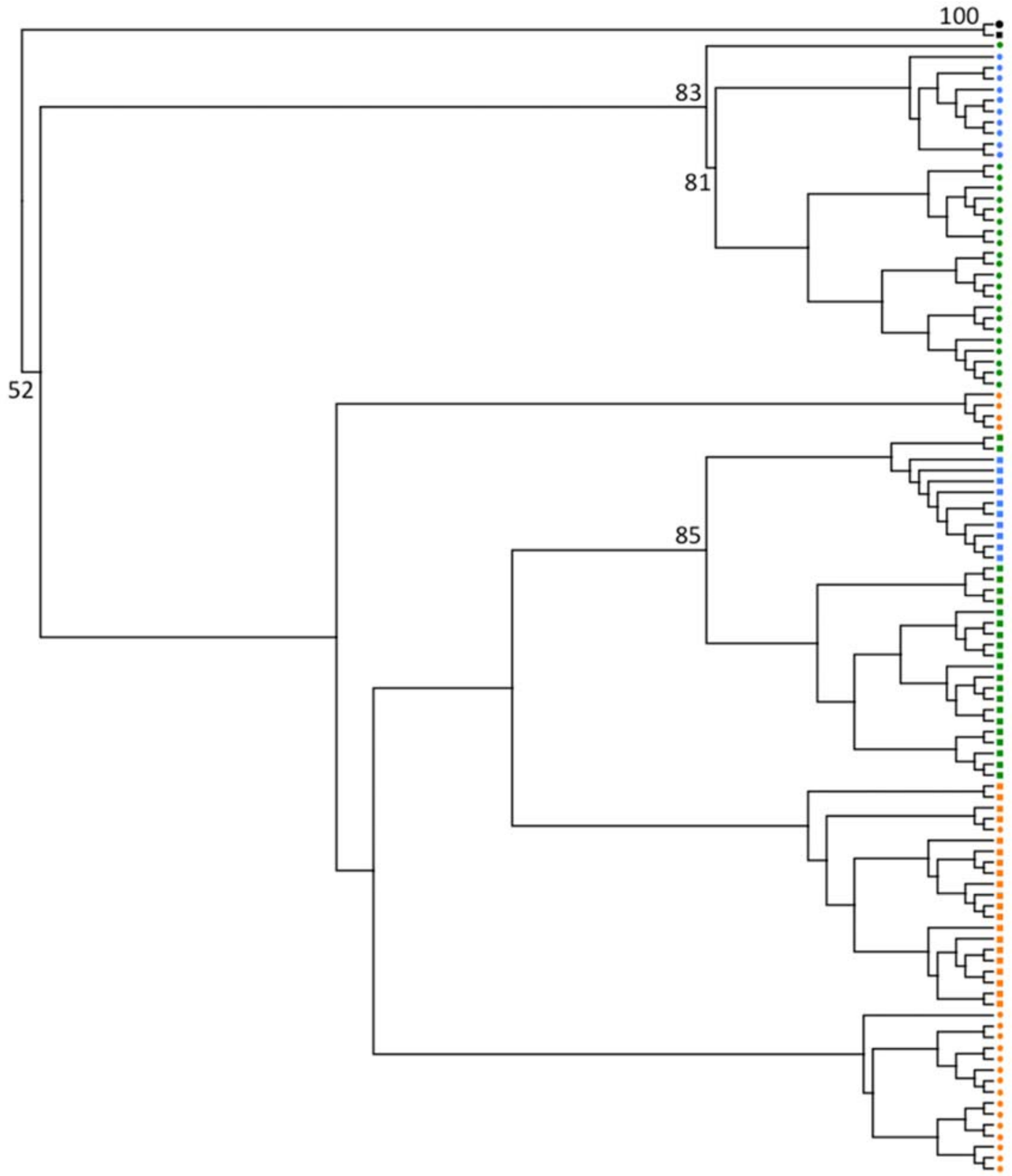

Unckless RL and Lazzaro B 2016. The potential for adaptive maintenance of diversity in insect antimicrobial peptides. *Phil. Trans. R. Soc. B.* doi:10.1098/rstb.2015.0291

Figure S2. Convergence of TSPs on the *Drosophila* phylogeny. Letters represent amino acids at the position along the top row (indicated for each gene – see Table 1). We considered polymorphism data for *D. mauritiana*, *D. simulans*, *D. mauritiana* and *D. yakuba.* Segregating amino acids are indicated by consecutive numbers. Amino acids in close paralogs are separated by a slash. Dashes represent data unavailable due to poor alignments or lack of sequence. Colours are used to visually differentiate alleles.

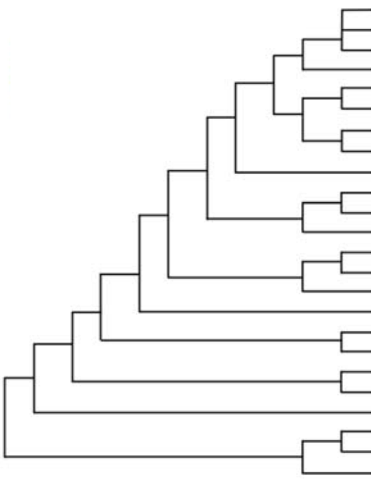

species	Mtk-m50	Dro-m52	DptB-sp19	DptA-m69	Def-sp17	Def-p20	CecA-s2	CecA-s17	CecB-s10	CecB-s15	AttC-p7	AttD-n40	AttD-n58	AttD-c3	AttD-c16	AttD-c85	AttAB-s9	AttAB-s15	AttAB-p1	AttAB-m8
D. mauritiana	RP	A	AV	S	MV	H	N	ST	VM	A	TN	AST	AG	MV	F	LV	LM/L	AV/A	LV/LV	A/A
D. simulans	RP	AT	A	SR	M	HQ	NK	T	VM	A	T	AST	AG	MV	FI	LV	LM/L	A/SA	LV/LV	AT/AT
D. sechellia	P	A	A	S	M	H	N	T	V	A	del	A	G	V	L	L	M	V	V	T
D. melanogaster	RP	AT	AV	SR	MV	H	NK	SAT	V	AV	TN	A	A	V	FI	L	V/L	AT/SAT	LV/LV	AT/AT
D. yakuba	RP	A	A	N	M	HQ	N	TA	V	AV	T	T	A	M	F	L	V/V	T/T	V/V	A/A
D. santomea	P	A	A	N	M	H	-	-	-	-	-	-	-	-	-	-	-	-	-	-
D. erecta	R	A	A	S	V	H	N	A	V	A	del	A	S	M	F	L	V/V	S/S	V/V	S/S
D. orena	R	S	A	R	M	H	N	A	V	A	T	A	A	M	F	L	V	-	-	-
D. eugracialis	P	V	A	S	M	Q	N	S	V	A	T	A	S	V	L	L	S	A/S	L/V	A/A
D. biarmipes	G	A	S	Q	L	H	N	S	V	A	T	T	G	V	L	L	V/V	S/S	V/V	A/A
D. suzukii	G	A	S	Q	-	H	N	S	V	A	T	-	G	V	H	L	V	T/T	V/V	A/A
D. takahashii	G	A	A	S	L	H	N	S	V	A	T	-	G	V	L	L	V	A/A	L/L	A/T
D. elegans	G	G	-	S	V	Q	N	S	V	A	T	T	G	M	L	L	V/V	T/T	F/F	A/A
D. rhopaloa	P	G	-	S	V	-	N	S	V	A	T	T	G	V	L	V	L/L	S/S	V/V	A/A
D. ficusphila	P	G	A	R	M	H	N	S	V	A	-	-	G	V	H	L	V	V	V	T
D. kikkawai	R	G	-	S	L	D	N	T	V	A	-	A	G	L	H	L	V/L	A/V	L/V	T
D. ananassae	P	G	A	Q	V	N	N	C	V	A	F	T	G	F	H	L	-	-	-	A
D. bipectinata	P	G	-	S	V	E	N	S	V	A	-	T	G	F	H	L	-	-	-	T
D. persimilis	P	G	-	S	L	D	-	-	-	-	-	T	G	I	L	L	V	A	V	A/A
D. pseudoobscura	P	G	-	S	L	D	N	S	V	A	-	T	G	I	L	L	V	G	V	A/A
D. willistoni	P	G	A	R	L	D	N	S	V	A	A	K	G	F	L	L	L/L	A/P	A/A	A/S
D. virilis	P	-	A	S	L	P	N	S	V	A	P	S	G	F	L	L	L/L	G/G	V/V	T/T
D. mojavensis	P	-	A	S	L/F	Q/L	N	S	V	A	-	T	G	S	F	V	L	G	V	S
D. grimshawi	P	-	A	S	-	-	-	-	V	A	T/N	V	S	F	F	V	L	A	I	S
